# Monte Carlo-based parametrization of the lateral dose spread for clinical treatment planning of scanned proton and carbon ion beams

**DOI:** 10.1093/jrr/rrt051

**Published:** 2013-07

**Authors:** Katia Parodi, Andrea Mairani, Florian Sommerer

**Affiliations:** 1 Heidelberg Ion Beam Therapy Center and Department of Radiation Oncology, Heidelberg University Clinic, Im Neuenheimer Feld 450, 69120 Heidelberg, Germany; 2 Present address: Ludwig Maximilian University of Munich, Am Coulombwall 1, 85748 Garching, Germany; 3 Present address: Centro Nazionale di Terapia Oncologica, Strada Campeggi, 53, 27100 Pavia, Italy

**Keywords:** ion therapy, Monte Carlo, treatment planning, lateral dose distribution

## Abstract

Ion beam therapy using state-of-the-art pencil-beam scanning offers unprecedented tumour-dose conformality with superior sparing of healthy tissue and critical organs compared to conventional radiation modalities for external treatment of deep-seated tumours. For inverse plan optimization, the commonly employed analytical treatment-planning systems (TPSs) have to meet reasonable compromises in the accuracy of the pencil-beam modelling to ensure good performances in clinically tolerable execution times. In particular, the complex lateral spreading of ion beams in air and in the traversed tissue is typically approximated with ideal Gaussian-shaped distributions, enabling straightforward superimposition of several scattering contributions. This work presents the double Gaussian parametrization of scanned proton and carbon ion beams in water that has been introduced in an upgraded version of the worldwide first commercial ion TPS for clinical use at the Heidelberg Ion Beam Therapy Center (HIT). First, the Monte Carlo results obtained from a detailed implementation of the HIT beamline have been validated against available experimental data. Then, for generating the TPS lateral parametrization, radial beam broadening has been calculated in a water target placed at a representative position after scattering in the beamline elements and air for 20 initial beam energies for each ion species. The simulated profiles were finally fitted with an idealized double Gaussian distribution that did not perfectly describe the nature of the data, thus requiring a careful choice of the fitting conditions. The obtained parametrization is in clinical use not only at the HIT center, but also at the Centro Nazionale di Adroterapia Oncologica.

## INTRODUCTION

In recent years the rapidly increasing application of light ion beams (protons and carbon ions) to external beam radiotherapy has been accompanied by new efforts in the development of commercial treatment-planning solutions capable of handling all the clinically available ion species in combination with state-of-the-art scanning beam delivery techniques. The latter accomplish 3D superimposition of individual Bragg-peaks by combining quasi-monoenergetic beam energy selection (using a passive degrader or at the accelerator level) with fast lateral magnetic deflection. This may enable utmost target-dose conformality for arbitrarily complex tumour geometries, at the expense of increased demands on the accuracy of the characterization and delivery of the individual pencil-like beams building up the integral dose distribution.

At the Heidelberg Ion Beam Therapy Center (HIT), intensity-controlled raster scanning [1] is the only employed beam application modality. Treatment planning is performed using the first commercial CE-labelled TPS (treatment-planning system) for ions Syngo PT Planning (Siemens AG), largely based on the research code TRiP98 that was developed and clinically used for the carbon ion pilot therapy project at the GSI Helmholtzzentrum für Schwerionenforschung in Darmstadt, Germany [2]. The analytical dose calculation engine relies on a pencil-beam algorithm combining laterally integrated depth–dose distributions in water, obtained from Monte Carlo calculations [3], with an analytical radial dose spread. The first clinically used version of the code only accounted for a single Gaussian lateral beam parametrization. This was assumed to be constant in depth for the less-scattering carbon ions similar to the GSI experience [2], while broadening in depth according to the formalism of [4] for the more scattering protons. However, a successive clinical release included an important upgrade to enable the so-called double Gauss parametrization, which was originally proposed by [5] for proton beams. Although not perfectly describing the nature of the beam [6], the second Gaussian component can account for the low-dose halo typically extending far beyond the central pencil-beam core. This halo originates from secondary particles or fragments produced in nuclear interaction, predominantly by energetic ions, or from primary particles undergoing large-angle scattering in the beamline components, especially for low energy protons [7]. Therefore, this work aimed at producing the parameters of the double Gauss parametrization to be input into the TPS, on the basis of Monte Carlo simulations including a realistic description of the HIT beamline.

## MATERIALS AND METHODS

The Monte Carlo computational environment based on the FLUKA code [8, 9], which was used for generation of the TPS laterally integrated basic data [3], was upgraded to more realistically account for the influence of the HIT beamline on lateral beam scattering. For this purpose, detailed proprietary information from the vendor was confidentially made available for generation of the exact nozzle geometry, overcoming the simplifications introduced in [3]. In particular, the vacuum window and the Beam and Application Monitoring System (BAMS, Siemens AG), consisting of three ionization chambers (ICs) and two multiwire proportional chambers (MWPCs), were modelled in detail in the FLUKA geometry. For this purpose, the replication (‘lattice’) capability of FLUKA was used in order to facilitate the reproduction of repetitive structures with only the definition of a basic element and the transformation rules of its repetition in space. This feature was especially convenient for modelling the large number of tungsten wires that are horizontally, vertically and obliquely (45°) aligned in each MWPC element. The initial beam dimension in vacuum was either deduced from available data acquired in the first MWPC, placed some centimetres after the vacuum window (for comparison to measured lateral profiles), or assumed to be symmetric and Gaussian-shaped with 2.5-mm full width at half maximum (for basic data generation). Validation of the implemented modelling was performed against experimental data of lateral beam broadening in air and water, which were collected in the extensive experimental campaign reported by [6]. Moreover, we verified the consistency of the resulting depth–dose distributions with the previously generated basic data using a simplified description of the HIT beamline (cf. Results).

The total lateral beam broadening modelled by the TPS can be described as σ^2^_tot_(*z*_eq_*, d*) = σ^2^_δ_ (*z*_eq_) + σ^2^_air_(*d*), where z_eq_ is the water-equivalent depth in the medium while *d* is the geometric depth in the room [6]. As σ_air_ is internally calculated by the TPS as a single Gaussian distribution mostly related to the beam focus in air at the isocenter extracted from the accelerator library [6], the sought double Gauss parametrization in condensed media σ_δ_ (*z*_eq_) for an initial infinitely narrow beam inherently includes the large-angle scattering contribution from the nozzle and the airgap. Since the latter depends on the target position in the treatment room, the choice was made by the medical physicists to have the Monte Carlo simulations performed for mono-energetic beams impinging on a water target with entrance surface shifted 12 cm upstream with respect to the treatment room isocenter. This was estimated to be a reasonable compromise, as the isocenter typically resides in the tumour, which is located downstream from the beam entrance in the patient. The Monte Carlo calculations were performed for 20 initial beam energies for each ion species under typical therapeutic conditions, i.e. with and without the ripple filter [3] for carbon ions and proton beams for a total of 3 × 10^6^ and 1.2 × 10^7^ primaries, respectively. The radial dose distribution *D*(*E,z*_*eq*_*,r*) was sampled with cylindrical scoring at 0.2 mm radial binning and in depth a finer separation of 0.18 mm (^12^C ions) or 0.2 mm (protons) in the Bragg peak, with a coarser grid of 2 mm elsewhere. The weighted least squares optimization fitting procedure was implemented in MATLAB 7.9.0 (The Mathworks Inc., USA) assuming an idealized double Gaussian distribution, aiming to deduce the global normalization factor *n* and the sigma values σ_1_ and σ_2_ of the narrow and broad Gaussians of unitary area with related weights (1 – *w*) and *w*, respectively:

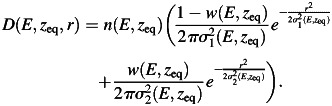



As the simulated distributions were extending at larger radial distances than the experimental values considered in [6], the fitting region was radially limited to enclose 99.73% of the total area of the radius-weighted distribution *r* · *D*(*E,z*_eq_*,r*). The most robust results were obtained when fitting the scored radial dose distribution *D*(*E,z*_eq_*,r*) with weights inversely proportional to *D*(*E,z*_eq_*,r*) for carbon ions, while fitting the radius-weighted *r* · *D*(*E,z*_eq_*,r*) distribution with weights inversely proportional to *r*^*2*^ · *D*(*E,z*_eq_*,r*) and keeping the σ_1_ value constant at depths beyond the 35% distal fall-off of the Bragg peak for protons. The resulting sigma parameters were finally corrected by quadratic subtraction of the initial beam width σ_air_(*E*,d) at the entrance to the water phantom, which was deduced from the fitted σ_1_ in the first cylindrical bin centred at the entrance air–water interface. For coupling to the TPS basic data, the penetration depth information *z*_eq_ was rescaled in such a way that depth 0 corresponded to the position before the vacuum window, while depth 1 corresponded to the Bragg peak position.

## RESULTS

**Fig. 1.**
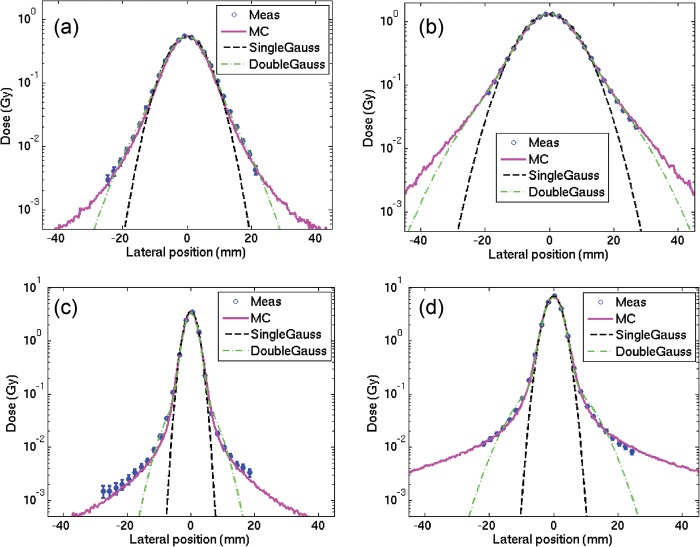
Example of measured lateral distributions [6] with corresponding MC simulations (normalized to the data) for proton (top, 157.53 MeV/u) and carbon ion (bottom, 299.94 MeV/u) beams in water, sampled at a depth of ∼1.5 cm in the entrance channel (left, **a** and **c**) and of ∼16.5 cm shortly before the Bragg peak (right, **b** and **d**). The double Gauss fit of the experimental data is also shown in comparison to the single Gauss approximation.

**Fig. 2.**
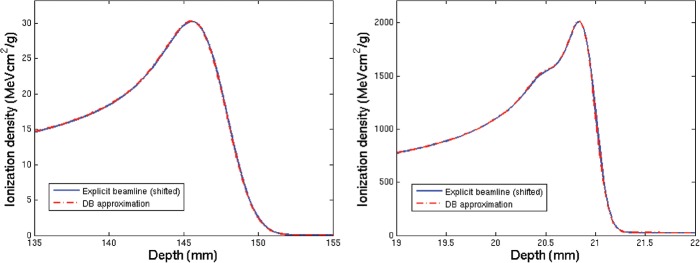
Example of laterally integrated depth–dose distributions obtained with the simplified description of the HIT beamline for initial basic data generation (‘DB approximation’) [3] in comparison with the distributions obtained with the upgraded geometry used in this work (‘Explicit beamline’) for an intermediate proton beam energy of 142.66 MeV/u (left) and the lowest carbon ion energy of 88.83 MeV/u (right), which is sensitive to the Bragg-peak modulation from the tungsten wires in the beamline. The introduced shift accounts for the range degradation in the elements of the beamline and in air, which is not modelled in the DB approximation.

**Fig. 3.**
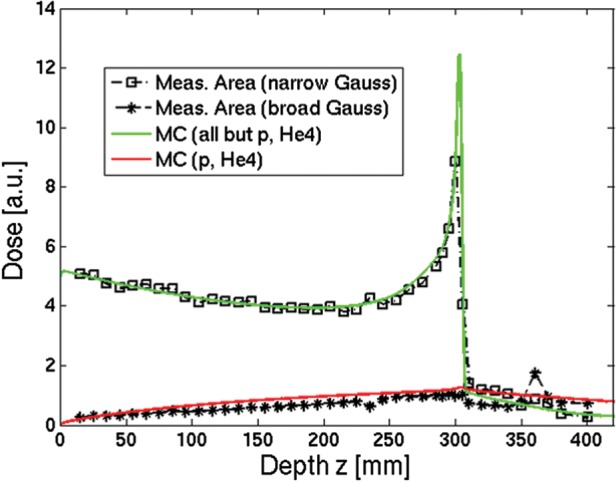
Separation in depth of the integral doses of the narrow (*n · (1 – w)*) and broad (*n · w*) components of the double Gauss parametrization of measured data [6] in comparison with the simulated laterally integrated dose contributions from the less-scattering (all but protons and alpha particles) and more-scattering (protons and alpha particles) primary and secondary ions produced by an energetic 430.10 MeV/u carbon ion beam in water.

The validation of the implemented simulation framework against experimental values measured for line scans of proton and carbon ion beams in water [6] is shown in semi-logarithmic scale in Fig. 1 for profiles sampled in the plateau and shortly before the Bragg peak region. A satisfactory agreement could be obtained, thus supporting the soundness of the new implementation of the beamline geometry. Moreover, the consistency between the resulting depth–dose distributions and the basic data previously calculated with a different description of the HIT beamline is shown in Fig. 2. It can be appreciated how the energy straggling due to the tungsten wires, only reproduced by a statistical approach in [3], is found to be in excellent agreement with the simulations including the explicit wire description. Finally, Fig. 3 shows the evolution in depth of the area of the two Gaussian components fitted on measured data for an energetic carbon ion beam in water. Interestingly, these data were found in good correspondence with the simulated dose contributions from secondary fragments and primary particles characterized by large-angle (protons and helium) as well as small-angle (heavier ions) scattering, respectively.

**Fig. 4.**
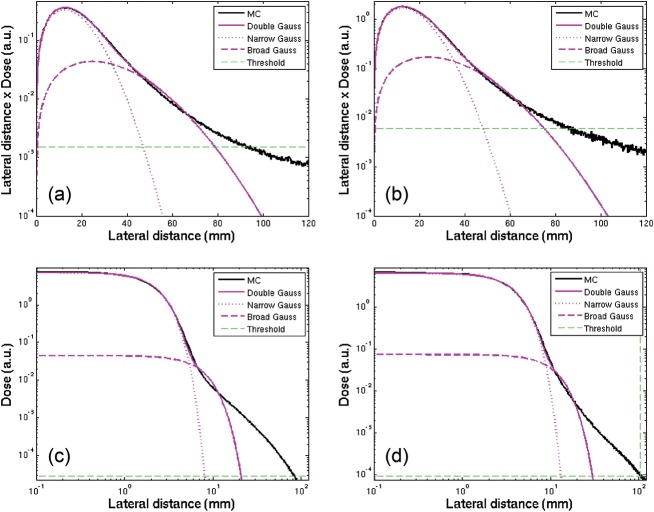
Example of simulated lateral profiles for basic data generation, with corresponding double Gauss fit for the proton lowest beam energy (48.12 MeV/u, top) and carbon ion highest beam energy (430.10 MeV/u, bottom) sampled at 20% (left, **a** and **c**) and 100% (right, **b** and **d**) of the Bragg peak position. The different representation reflects the different fitting of *r* · *D*(*E,z*_eq_*,r*) for protons (**a** and **b**) and *D*(*E,z*_*eq*_*,r*) for carbon ions (**c** and **d**), with related choice of semi- or double-logarithmic scale for improved display. Remaining limitations of the double Gaussian approximation are clearly visible, though mostly affecting the low-dose halo at a large distance from the central core of the pencil-beam. The green dashed line marks the level below which the data are no longer considered for the fit (cf. text).

**Fig. 5.**
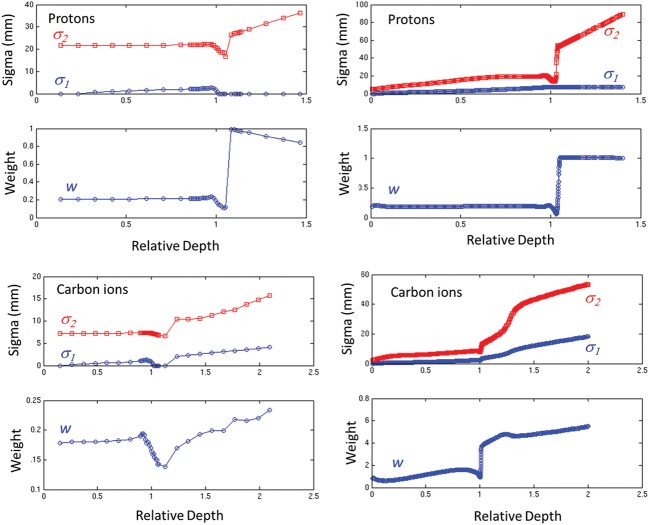
Typical trends in depth of the lateral basic data (i.e. fitted σ_1_ and σ_2_ and weight parameter *w* of the double Gauss) for protons (top) and carbon ions (bottom) at the lowest (left) and highest (right) available beam energy.

**Fig. 6.**
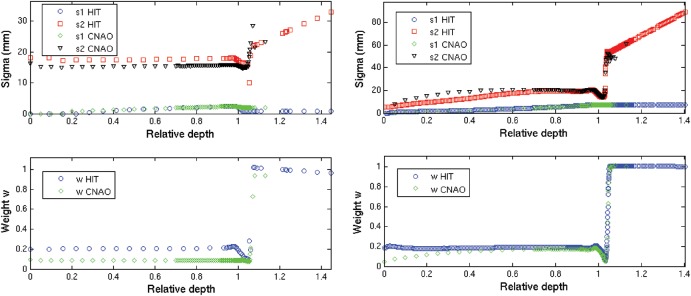
Comparison of the HIT basic data input in both the HIT and CNAO TPS, with respect to the parameters deduced using the same fitting procedure described in this work, however applied to FLUKA MC simulations of lateral beam broadening in water using a detailed modelling of the CNAO beamline for proton beams [11]. Two similar energies of approximately 60 MeV/u (left) and 221 MeV/u (right) were considered. While the sigma parameters match fairly well, some discrepancies are observed in the weight factor *w* directly related to the broad Gaussian component, especially for low-energy proton beams. This is likely ascribed to less large-angle scattering material in the CNAO beamline.

An illustration of the fitting results against the simulated values for basic data generation is shown in Fig. 4, highlighting unavoidable limitations of the double Gaussian parametrization for both proton and carbon ion beams. In particular, the ratio between the areas encompassed by the fitted and simulated lateral profiles varied from a few permille or percent at the entrance channel to up to 10–45% after the Bragg peak for protons (with the largest deviations for the lower energies) and up to 5–15% around the Bragg peak for carbon ions (with the largest deviations for the higher energies), however mostly affecting the loss of out-of-axis dose contribution at large radial distances. The typical behaviour of the obtained double Gaussian parametrization in depth is depicted in Fig. 5. The generated data have been in clinical use at the Heidelberg Ion Beam Therapy Center since summer 2011. The considerable improvement in the agreement between TPS calculations and dosimetric measurements in water has been recently reported [10]. Moreover, the data have also been used for clinical treatment at the Centro Nazionale di Adroterapia Oncologica (CNAO) since the beginning of its operation, also yielding satisfactory agreement in terms of dosimetric treatment plan verification [11], despite having been generated for a different beamline, resulting in the differences quantified in Fig. 6.

## DISCUSSION

This paper has described the work carried out to generate the double Gauss parametrization for the Syngo PT TPS, which is in clinical use at HIT and CNAO for proton and carbon ion beams. The detailed implementation of the HIT beamline in the FLUKA code could reasonably reproduce lateral beam broadening in air and water measured at representative beam energies and depths, thus justifying its usage for generation of the TPS basic data. This strategy offers higher granularity and reduced measuring time in comparison to a purely experimental approach.

Although the idealized double Gaussian distribution does not perfectly describe the nature of the data, the Monte Carlo-based parametrization input into the commercial TPS clearly provides an improvement over the initial single Gaussian approximation. So far, good agreement typically within 2–3% has been independently reported by the medical physics teams of the two facilities in Heidelberg and Pavia for dosimetric measurements and TPS calculations in water, when using the same double Gaussian parametrization optimized for the HIT beamline. Moreover, improved agreement is also observed between the TPS predictions and the full Monte Carlo recalculations of patient treatments in CT (computed tomography) geometry, as being pursued with the dedicated computational framework developed within the FP7 PARTNER Project [12].

## FUNDING

This work was supported by the Seventh Framework Programme FP7/2007–2013 PARTNER (Grant Agreement No. 215840-2 to FS).
